# Cognitive flexibility mediates the impact of emotion regulation strategies on negative emotions in preschool teachers

**DOI:** 10.3389/fpsyg.2025.1609872

**Published:** 2025-07-16

**Authors:** Changwei Gu, Mengxin Guo, Yeying Cui, Feifei Yu, Ya Chen, Juanjuan Chu, Shuang Zhou

**Affiliations:** ^1^College of Preschool Education, Capital Normal University, Beijing, China; ^2^Suzhou Education Quality Monitoring Centre, Suzhou, China; ^3^Suzhou New District Experimental Primary School, Suzhou, China; ^4^Beijing National Day Jin Yuan Kindergarten, Beijing, China; ^5^College of Physical Education, Shanxi Vocational University of Engineering Science and Technology, Jinzhong, China

**Keywords:** emotion regulation, cognitive flexibility, preschool teachers, anxiety, depression

## Abstract

This study aimed to examine how cognitive flexibility mediates the associations between emotion regulation strategies and negative emotions among preschool teachers in China. A total of 392 in-service preschool teachers in Beijing were recruited through random sampling. Participants completed validated questionnaires assessing cognitive reappraisal and expressive suppression (Emotion Regulation Questionnaire), anxiety and depression (SAS and SDS), and cognitive flexibility (Cognitive Flexibility Inventory). Structural equation modeling (SEM) and bootstrapping were used to test mediation models. The results revealed that cognitive reappraisal positively predicted cognitive flexibility, which in turn was associated with lower levels of both anxiety and depression. Conversely, expressive suppression negatively predicted cognitive flexibility, which indirectly contributed to increased negative emotions. However, the direct effects of expressive suppression on anxiety and depression were not statistically significant. These findings suggest that cognitive flexibility serves as a crucial psychological mechanism through which emotion regulation strategies impact mental health. This study advances current theoretical models by highlighting cognitive flexibility as a mediating factor in preschool teachers’ emotional experiences.

## 1 Introduction

Preschool teachers routinely engage in extensive emotional labor due to the nature of their professional roles, which involve continuous interactions with young children, colleagues, and parents, often requiring effective management of their emotional states (Jeon et al., 2016; Wang et al., 2019). Given these constant emotional demands, preschool teachers are susceptible to experiencing elevated levels of negative emotions, such as anxiety and depression, significantly impacting their psychological health and professional effectiveness (Jeon et al., 2018). Emotion regulation strategies, particularly cognitive reappraisal and expressive suppression, play a critical role in shaping teachers’ emotional experiences and coping capacities (Gross, 2024; Lee et al., 2016). Effective emotion regulation can mitigate negative emotional experiences, thus preserving mental health and enhancing overall job performance (Buruck et al., 2016; Peng et al., 2019; Wang et al., 2023). Recent research underscores cognitive flexibility as a key psychological factor that facilitates adaptive emotion regulation, allowing individuals to respond flexibly and constructively to changing emotional demands (Gao et al., 2025; Pruessner et al., 2020). Despite growing attention to emotion regulation among educators, the mediating role of cognitive flexibility, especially within the emotionally demanding context of preschool education, remains underexplored. As mediators in children’s socialization, preschool teachers’ emotional regulation strategies, embedded in daily interactions, shape emotional cognition and foster prosocial behaviors. Therefore, examining how cognitive flexibility mediates the relationship between specific emotion regulation strategies and negative emotional outcomes among preschool teachers is both theoretically valuable and practically imperative.

Emotion regulation has increasingly become a critical area of investigation within emotional psychology, particularly in the context of occupational health. Emotion regulation strategies typically include cognitive reappraisal, which involves actively reinterpreting emotionally charged situations in less distressing ways, and expressive suppression, which involves consciously inhibiting emotional expressions (Gross, 1998, 2015a, 2024). Prior studies have extensively documented differential outcomes associated with these strategies: cognitive reappraisal is consistently associated with better psychological health outcomes such as reduced anxiety and depression, whereas expressive suppression has typically been correlated with heightened emotional distress and poorer psychological well-being (Haga et al., 2009; Morrish et al., 2017). Previous studies found that teachers who frequently employed cognitive reappraisal reported lower job-related emotional exhaustion compared to those who habitually relied on expressive suppression (Taxer et al., 2019), indicating the adaptive benefits of cognitive reappraisal in professional contexts.

Although substantial empirical evidence demonstrates the direct relationships between emotion regulation strategies and emotional outcomes, emerging literature suggests the importance of investigating psychological mechanisms that explain these associations. Spiro et al. (1991) proposed cognitive flexibility theory, emphasizing multidimensional understanding and adaptive knowledge application in complex contexts. Cognitive flexibility—defined as the capacity to adaptively shift cognitive sets, perspectives, or approaches when facing changing situational demands—has recently gained prominence as a potential explanatory mechanism underlying adaptive emotional responses (Guassi Moreira et al., 2022; Pruessner et al., 2020). Research has shown cognitive flexibility to be positively correlated with emotional resilience and psychological well-being, emphasizing its role as an essential psychological resource in managing occupational stressors (Aldao et al., 2015; Bonanno and Burton, 2013). Studies highlighted cognitive flexibility as a significant predictor of lower depressive symptoms in educators, suggesting that cognitively flexible individuals are better equipped to navigate emotionally challenging professional environments (Deveney and Deldin, 2006).

Nevertheless, despite the growing recognition of cognitive flexibility as a protective factor, limited research explicitly explores how cognitive flexibility mediates the relationship between specific emotion regulation strategies and negative emotional outcomes within the preschool teaching profession. Preschool teachers represent a distinctive occupational group characterized by intensive emotional labor, as they are frequently required to regulate emotions to maintain positive interactions and classroom climates (Peng et al., 2019; Purper et al., 2023; Zhang et al., 2020). Previous studies have predominantly focused on direct associations between emotion regulation strategies and teacher well-being or job outcomes without adequately addressing underlying psychological mechanisms such as cognitive flexibility (Jeon et al., 2018; Wang et al., 2023). Thus, it remains unclear how cognitive flexibility influences the effectiveness of emotion regulation strategies—such as cognitive reappraisal and expressive suppression—in reducing negative emotions among preschool educators.

Although existing literature has significantly advanced our understanding of emotion regulation strategies and their influence on psychological well-being in various professional contexts, several critical research gaps persist. Firstly, previous research has predominantly emphasized the direct associations between emotion regulation strategies—particularly cognitive reappraisal and expressive suppression—and emotional outcomes such as anxiety and depression (Cutuli, 2014; Dryman and Heimberg, 2018). However, limited attention has been given to uncovering the mediating psychological processes that may elucidate why certain emotion regulation strategies effectively reduce negative emotions, whereas others do not. This limitation hampers the development of targeted psychological interventions designed to bolster emotional resilience and mental health among educators. Secondly, while cognitive flexibility has been increasingly recognized as a potentially critical mediator in the relationship between emotion regulation and emotional well-being (Deveney and Deldin, 2006; Gao et al., 2025; Motevalli et al., 2023), empirical research explicitly testing this mediation model in the context of preschool teachers remains notably scarce. Preschool teachers constitute a unique occupational group with high emotional labor demands, making it particularly relevant and necessary to identify the mechanisms that promote adaptive emotion regulation in their professional environment (Peng et al., 2019; Zhang et al., 2020). Therefore, elucidating how cognitive flexibility mediates the relationship between emotion regulation strategies and negative emotional outcomes in this specific professional group represents a significant and timely research priority.

To address these gaps, the current study employs Gross’s Process Model of Emotion Regulation (Gross, 2015b), integrating it with recent theoretical advancements regarding cognitive flexibility as an adaptive psychological mechanism (Aldao et al., 2015; Bonanno and Burton, 2013; Pruessner et al., 2020). Consequently, the primary objectives of the present research are twofold: first, to investigate the direct effects of cognitive reappraisal and expressive suppression on preschool teachers’ negative emotional outcomes (anxiety and depression); and second, to explore the mediating role of cognitive flexibility in these relationships. Specifically, we hypothesize that:

*H1*: Cognitive reappraisal is hypothesized to positively predict cognitive flexibility among preschool teachers, whereas expressive suppression is expected to negatively predict cognitive flexibility.

*H2*: Cognitive flexibility is hypothesized to negatively predict negative emotional outcomes, including anxiety and depression.

*H3*: Cognitive flexibility is hypothesized to mediate the relationship between emotion regulation strategies (cognitive reappraisal and expressive suppression) and negative emotional outcomes.

**Figure 1:**
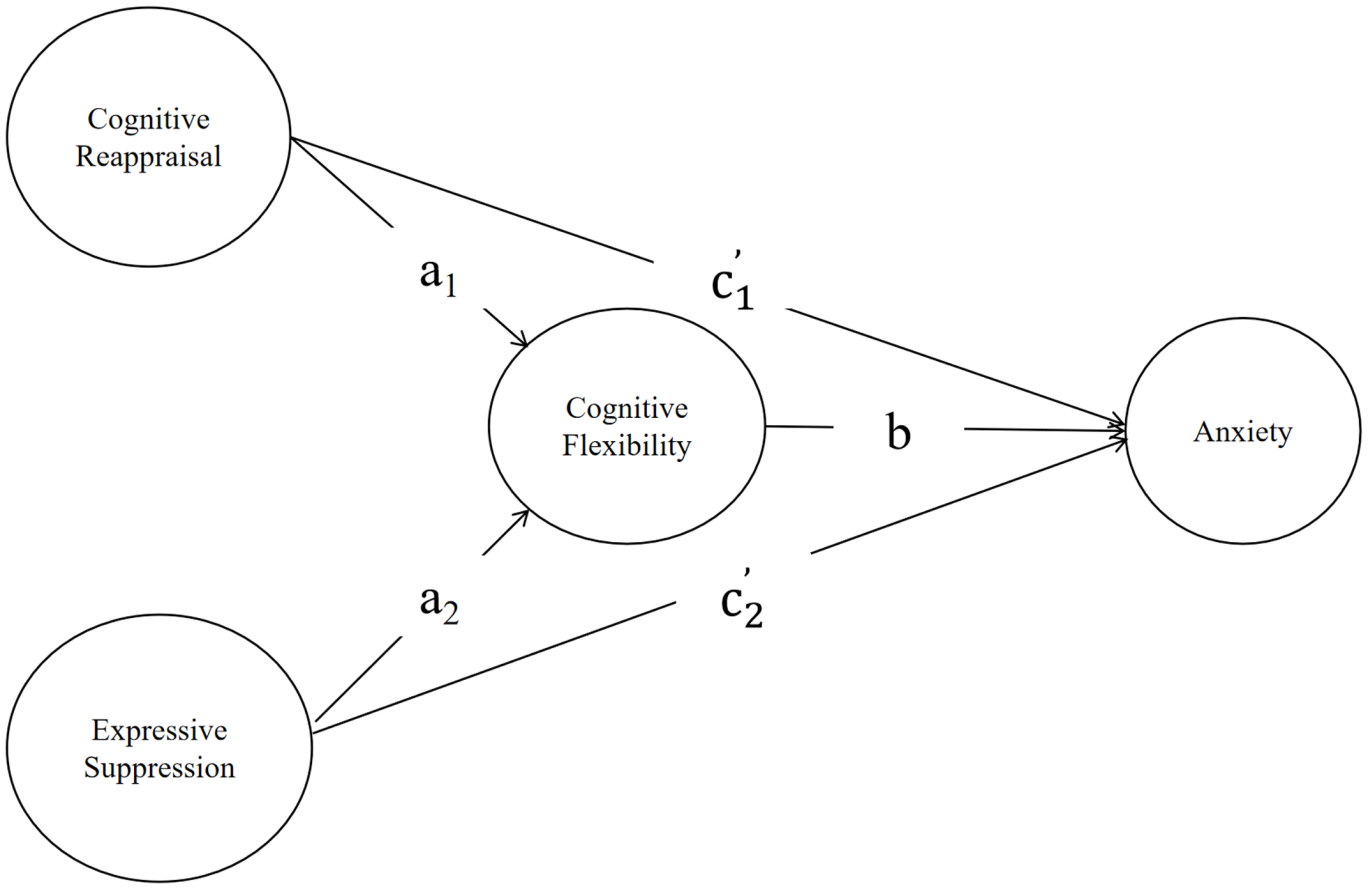
The hypothetical model of anxiety.

The hypothesized models are as follows:

Model 1 (Anxiety): Emotion regulation strategies (cognitive reappraisal and expressive suppression) affect anxiety through cognitive flexibility (Figure 1). (1) the indirect effect of cognitive reappraisal on anxiety via cognitive flexibility (i.e., a_1_ × b), and the direct effect of cognitive reappraisal on anxiety is c_1_’; (2) the indirect effect of expressive suppression on anxiety via cognitive flexibility (i.e., a_2_ × b), and the direct effect of expressive suppression on anxiety is c_2_’.

**Figure 2:**
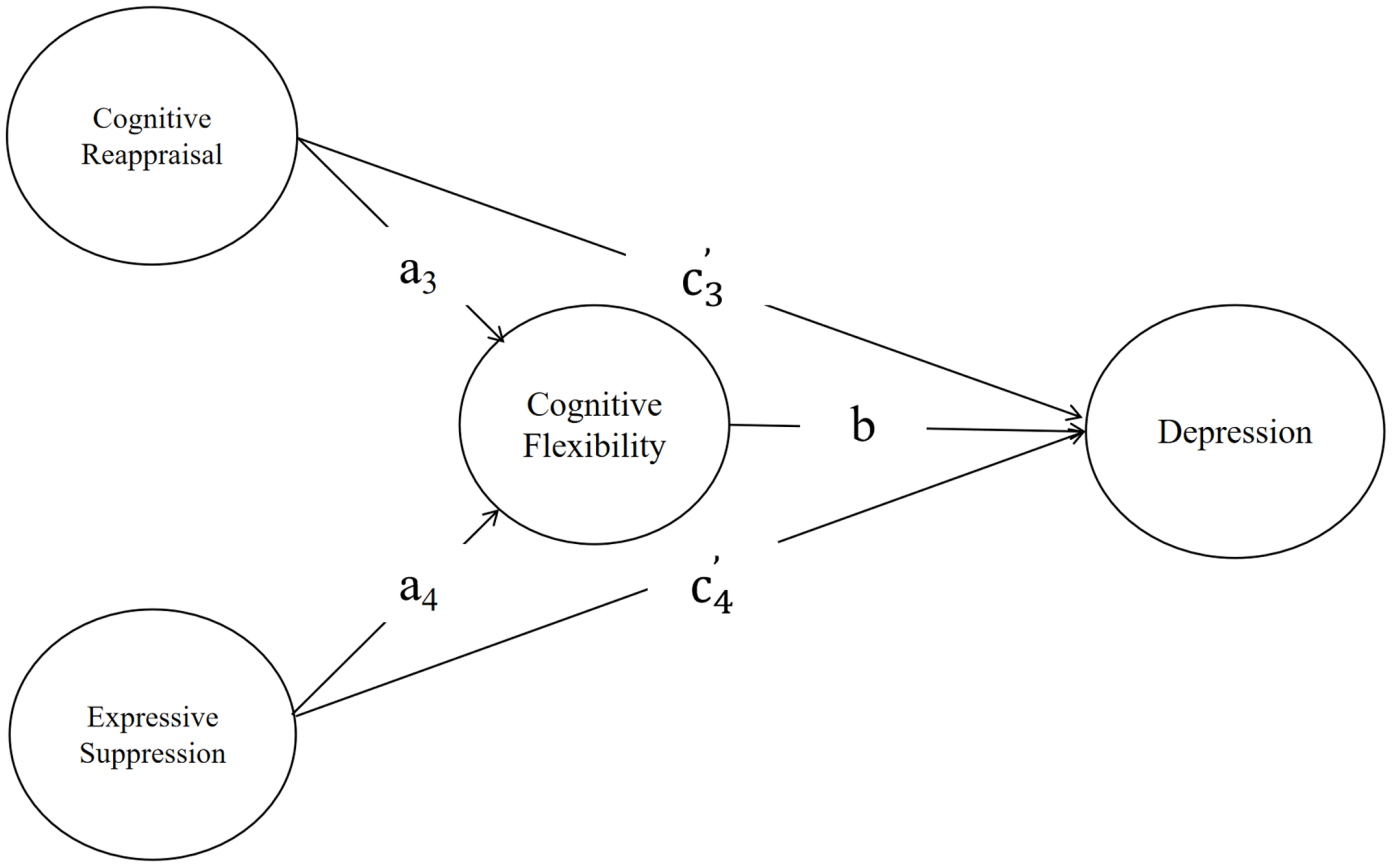
The hypothetical model of depression.

Model 2 (Depression): Emotion regulation strategies (cognitive reappraisal and expressive suppression) affect depression through cognitive flexibility (Figure 2). (1) the indirect effect of cognitive reappraisal on depression via cognitive flexibility (i.e., a3 × b), and the direct effect of cognitive reappraisal on depression is c3’; (2) the indirect effect of expressive suppression on depression via cognitive flexibility (i.e., a4 × b), and the direct effect of expressive suppression on depression is c4’.

## 2 Methods

### 2.1 Participants

**Figure 3:**
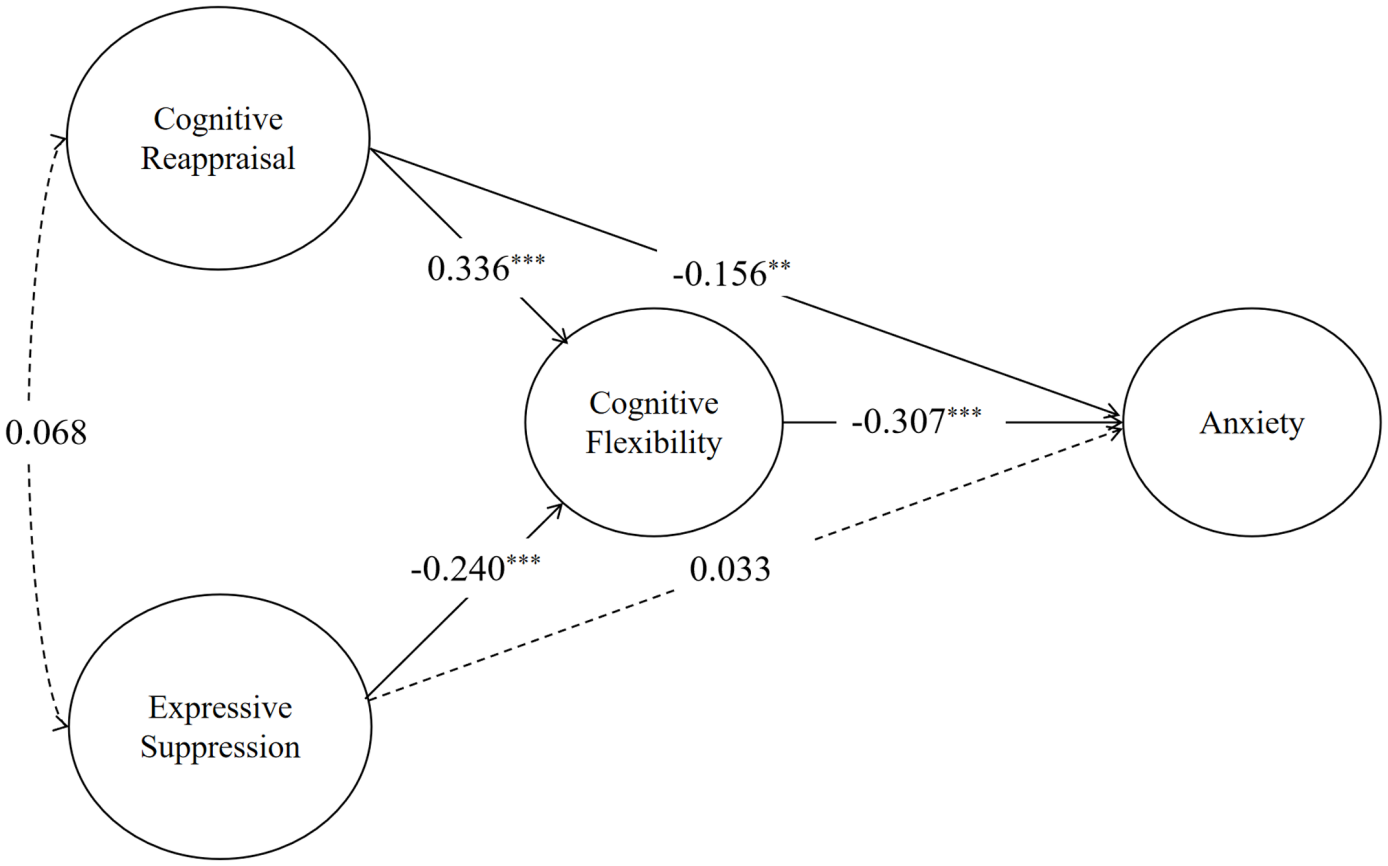
SEM model showing the mediating effect of cognitive flexibility between emotion regulation strategies and anxiety. Solid lines indicate significant paths (*p* < 0.05), while dashed lines indicate non-significant paths.

Sample size estimation for this study was guided by mediation effect testing requirements, using Fritz and MacKinnon’s (2007) method for mediation models. Ultimately, through simple random sampling, 400 questionnaires were distributed among 10 kindergartens in Beijing, and a total of 392 valid questionnaires were collected, resulting in an effective response rate of 98%. This sample size was sufficient for analyzing the complex mediation framework, and the study was approved by the ethics committee of the College of Preschool Education, Capital Normal University (Figure 3).

### 2.2 Rating scales

#### 2.2.1 Questionnaire on emotion regulation strategies

**Figure 4:**
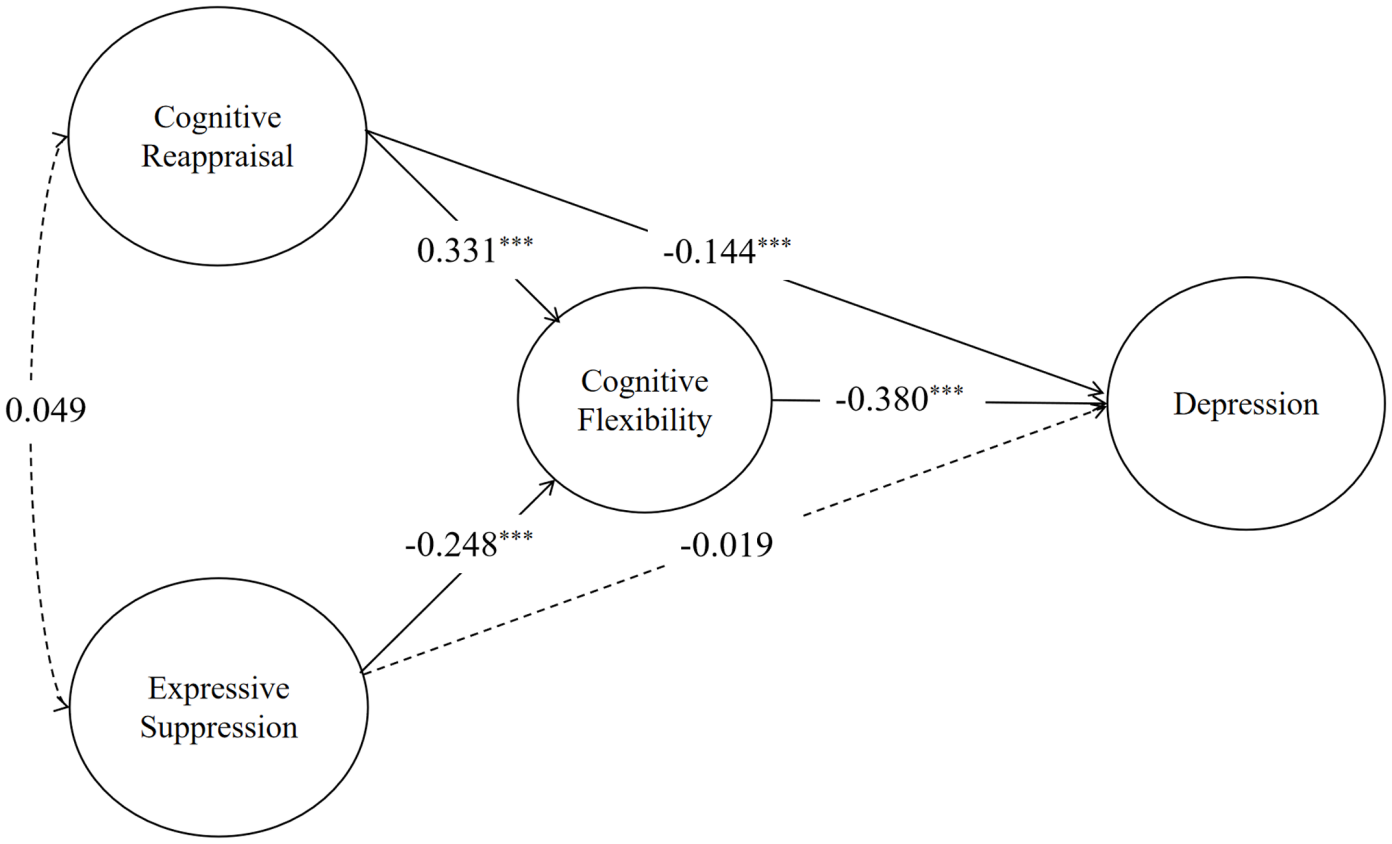
SEM model showing the mediating effect of cognitive flexibility between emotion regulation strategies and depression. Solid lines indicate significant paths (*p* < 0.05), while dashed lines indicate non-significant paths.

The Emotion Regulation Questionnaire (ERQ) developed by Gross and John (2003a,b) was used with a total of 10 items, including two dimensions: cognitive reappraisal and expressive suppression. The questionnaire is scored on a seven-point scale from 1 (strongly disagree) to 7 (completely agree), with higher scores indicating more frequent use of emotion regulation strategies. In this study, the Cronbach’s alpha was 0.852 (Figure 4).

#### 2.2.2 Mental health questionnaire

##### 2.2.2.1 Self-rating anxiety scale (SAS)

This scale was developed by Zung (1971) and consists of 20 items, each rated on a 4-point Likert scale the total score of the 20 items is multiplied by 1.25 and rounded to obtain the final anxiety score. A score below 50 is considered normal, 50–59 indicates mild anxiety, 60–69 indicates moderate anxiety, and 70 and above indicates severe anxiety. In this study, a cutoff score of ≥50 was used to identify preschool teachers experiencing anxiety. Higher SAS scores indicate more severe anxiety levels. The Cronbach’s alpha coefficient for this scale in the current study was 0.76.

##### 2.2.2.2 SDS Self-rating depression scale (SDS)

This scale was developed by Zung (1965) and consists of 20 items. Each item is rated on a 4-point Likert scale based on feelings experienced in the past week. The total score is calculated by summing the item scores, and the final depression score is obtained using the formula: Depression Score = Total Item Score / 80. A score below 0.50 indicates no depression, 0.50–0.59 indicates mild to moderate depression, 0.60–0.69 indicates moderate to severe depression, and 0.70 and above indicates severe depression. In this study, the Cronbach’s alpha coefficient for this scale was 0.841.

#### 2.2.3 Cognitive flexibility questionnaire

The Chinese version of the Cognitive Flexibility Inventory (CFI) was used in this study (Wang, 2016). The scale consists of 20 items rated on a 5-point Likert scale ranging from “never” to “always,” with six items being reverse-scored. The total score is obtained by summing the scores of all dimensions, with higher scores indicating greater cognitive flexibility. In this study, the total score was used to measure cognitive flexibility, and the Cronbach’s alpha coefficient for this scale was 0.841.

### 2.3 Statistical methods

Mplus 8 software was used to construct a multiple mediation model through structural equation modeling (Preacher and Hayes, 2008). Mediation effects were estimated using the bias-corrected percentile Bootstrap method with 5,000 resampling iterations to calculate 95% confidence intervals (CIs). A two-tailed test was conducted, with statistical significance set at *p* < 0.05.

### 2.4 Common method bias test

In the questionnaire design and distribution process, this study employed proactive controls to mitigate common method bias, including anonymous response collection and the inclusion of reverse-coded items. Common method bias was evaluated using Harman’s single-factor test, which extracted 13 factors with eigenvalues greater than 1. The first factor accounted for 25.44% of the explained variance, falling below the 40% critical threshold. These findings indicate that the study’s data were not seriously compromised by common method bias.

## 3 Results

### 3.1 Descriptive statistics and correlation analysis

The descriptive statistics and correlation analysis are presented in Table 1. The results indicate that cognitive reappraisal is significantly negatively correlated with anxiety and depression, and significantly positively correlated with cognitive flexibility. Conversely, expressive suppression is significantly positively correlated with anxiety and depression, while showing a significant negative correlation with cognitive flexibility. Additionally, anxiety scores are significantly positively correlated with depression levels and significantly negatively correlated with cognitive flexibility. Depression scores are also significantly negatively correlated with cognitive flexibility (Table 2).

**Table 1:** Analysis of demographic variables of participants.

Variables	Background variables	*N*	Percentage
Gender	Male	16	4.08%
	Female	376	95.92%
Age	Under 20 years old	11	2.81%
	21–25 years old	111	28.32%
	26–30 years old	125	31.89%
	Over 31 years old	145	36.99%
Education Level	High school or secondary school and below	54	13.78%
	Associate degree	140	35.71%
	Bachelor Degree	167	42.60%
	Master’s degree or above	31	7.91%
Teaching Experience	0–1 year	132	33.67%
	1–3 years	99	25.26%
	3-5 years	72	18.37%
	Over 5 years	89	22.70%
Position	Head Teacher	132	33.67%
	Assistant Teacher	161	41.07%
	Daycare Worker	74	18.88%
	Administrative Staff	25	6.38%

**Table 2:** Descriptive statistics and correlation analysis of research variables.

Variable	M ± SD	1	2	3	4
1. Cognitive Reappraisal	5.15 ± 1.08				
2. Expressive Suppression	3.68 ± 1.25	0.06			
3. Anxiety	47.65 ± 11.11	−0.39^**^	0.11^*^		
4. Depression	0.49 ± 0.12	−0.48^***^	0.20^*^	0.45^**^	
5. Cognitive Flexibility	3.53 ± 0.56	0.54^***^	−0.32^**^	−0.45^***^	−0.67^***^

^*^p < 0.05, ^**^p < 0.01, ^***^p < 0.001.

### 3.2 Measurement models and hypothetical models

To assess the measurement model, we conducted a confirmatory factor analysis (CFA), including variables such as cognitive reappraisal, expressive suppression, anxiety, depression, and cognitive flexibility. The CFA results indicated a satisfactory model fit: *χ*^2^/df = 2.202 (*χ*^2^ = 284.97, *df* = 84, *p* < 0.001), RMSEA = 0.06, CFI = 0.96, TLI = 0.95, SRMR = 0.05, indicating that the model structure was appropriate for further analysis.

Based on the correlation analysis, we developed two structural equation models (SEMs). Considering that anxiety and depression represent distinct psychological outcomes potentially influenced differently by emotion regulation strategies, we modeled them separately to achieve more accurate assessments. Model 1 examined the relationship between emotion regulation strategies and anxiety, with cognitive flexibility as a mediator. Model 2 focused on the relationship between emotion regulation strategies and depression, also mediated by cognitive flexibility. In both models, emotion regulation strategies were decomposed into two dimensions: cognitive reappraisal and expressive suppression, consistent with the ERQ questionnaire framework.

### 3.3 Structural equation model path analysis

The SEM path analysis for Anxiety indicate that cognitive reappraisal has a significant positive effect on cognitive flexibility (*β* = 0.627, *p* < 0.001). In contrast, expressive suppression has a significant negative effect on cognitive flexibility (*β* = −0.426, *p* < 0.001). Additionally, cognitive flexibility shows significant negative effects on anxiety, with standardized coefficients of −0.311.

The indirect effect of cognitive reappraisal on anxiety through cognitive flexibility was significant (*β* = −0.195, *p* < 0.01), as was the direct effect on anxiety (*β* = −0.294, *p* < 0.01). The indirect effect of expressive suppression on anxiety through cognitive flexibility was significant (*β* = 0.132, *p* < 0.01). However, expressive suppression demonstrates a non-significant positive direct effect on anxiety, with a standardized coefficient of 0.060 (Table 3).

**Table 3:** Results of SEM and bootstrapping for anxiety.

Effect	Unstd. Coeff	Std. Coeff.	S. E.	C. R.	*p*
Cognitive Reappraisal → Cognitive Flexibility	0.336	0.627	0.037	16.787	<0.001
Expressive Suppression → Cognitive Flexibility	−0.240	−0.426	0.073	−5.804	<0.001
Cognitive Flexibility → Anxiety	−0.307	−0.311	0.086	−3.636	<0.001
Cognitive Reappraisal → Anxiety (Total)	−0.259	−0.259	0.048	−5.344	< 0.001
Cognitive Reappraisal → Anxiety (Direct)	−0.156	−0.294	0.106	−2.779	< 0.01
Cognitive Reappraisal → Cognitive Flexibility → Anxiety (Indirect)	−0.103	−0.195	0.028	−3.630	< 0.01
Expressive Suppression → Anxiety (Total)	0.107	0.1	0.026	4.083	< 0.001
Expressive Suppression → Anxiety (Direct)	0.033	0.060	0.070	0.855	0.392
Expressive Suppression → Cognitive Flexibility → Anxiety (Indirect)	0.074	0.132	0.026	2.870	< 0.01

^*^p < 0.05, ^**^p < 0.01, ^***^p < 0.001.

Same analysis on depression indicate that cognitive reappraisal has a significant positive effect on cognitive flexibility (*β* = 0.609, *p* < 0.001). In contrast, expressive suppression exhibits a significant negative effect on cognitive flexibility (*β* = −0.429, *p* < 0.001). Furthermore,cognitive flexibility demonstrate significant negative impact on depression, with standardized coefficients of −0.366.

The indirect effect of cognitive reappraisal on depression through cognitive flexibility was significant (*β* = −0.223, *p* < 0.01), as was the direct effect on depression (*β* = −0.032, *p* < 0.01). The indirect effect of expressive suppression on anxiety through cognitive flexibility was significant (*β* = 0.157, *p* < 0.01), as was the direct effect on anxiety (*β* = −0.255, *p* < 0.001) (Table 4).

**Table 4:** Results of SEM and bootstrapping for depression.

Effect	Unstd. Coeff	Std. Coeff.	S. E.	C. R.	*p*
Cognitive Reappraisal → Cognitive Flexibility	0.331	0.609	0.047	12.920	<0.001
Expressive Suppression → Cognitive Flexibility	−0.248	−0.429	0.050	−8.545	<0.001
Cognitive Flexibility → Depression	−0.380	−0.366	0.064	−5.744	<0.001
Cognitive Reappraisal → Depression (Total)	−0.269	−0.269	0.038	−7.045	< 0.001
Cognitive Reappraisal → Depression (Direct)	0.049	−0.032	0.082	−0.391	0.696
Cognitive Reappraisal → Cognitive Flexibility → Depression (Indirect)	−0.126	−0.223	0.028	−4.480	< 0.001
Expressive Suppression → Depression (Total)	0.075	0.075	0.047	1.593	0.111
Expressive Suppression → Depression (Direct)	−0.144	−0.255	0.058	−4.381	< 0.001
Expressive Suppression → Cognitive Flexibility → Depression (Indirect)	0.094	0.157	0.023	4.028	< 0.001

^*^p < 0.05, ^**^p < 0.01, ^***^p < 0.001.

## 4 Discussion

The overarching hypothesis of the present study was that cognitive flexibility mediates the relationships between emotion regulation strategies (cognitive reappraisal and expressive suppression) and negative emotional outcomes (anxiety and depression) among preschool teachers. Using structural equation modeling, cognitive reappraisal was positively associated with cognitive flexibility, which, in turn, negatively predicted both anxiety and depression. In contrast, expressive suppression showed a significant negative association with cognitive flexibility, indirectly contributing to higher levels of negative emotions, although its direct effects on anxiety and depression were minimal and non-significant. These results provide robust empirical evidence supporting cognitive flexibility as an essential mediator in understanding how emotion regulation strategies impact preschool teachers’ experiences of negative emotions.

The most significant finding of this study is the mediating role of cognitive flexibility between emotion regulation strategies—cognitive reappraisal and expressive suppression—and negative emotional outcomes (anxiety and depression) among preschool teachers. Specifically, cognitive reappraisal positively predicted cognitive flexibility, subsequently leading to decreased anxiety and depression. This finding aligns with our hypothesis and previous research highlighting cognitive reappraisal as an adaptive emotion regulation strategy linked with improved psychological health and resilience (Dryman and Heimberg, 2018; Stover et al., 2024; Troy et al., 2010). Moreover, present results indicates that cognitive reappraisal helps individuals reinterpret emotional experiences positively, thereby enhancing adaptive cognitive functioning such as cognitive flexibility. Thus, our findings extend existing knowledge by elucidating the underlying mechanism—cognitive flexibility—that contributes to the effectiveness of cognitive reappraisal in mitigating negative emotional experiences in an occupational setting characterized by intensive emotional labor.

Conversely, expressive suppression demonstrated a significant negative relationship with cognitive flexibility, indirectly contributing to increased anxiety and depression among preschool teachers. While the direct effect of expressive suppression on anxiety and depression was minimal and non-significant, the negative association with cognitive flexibility suggests a maladaptive cognitive pattern fostered by suppressing emotional expressions. Previous studies consistently report expressive suppression as a maladaptive regulation strategy associated with higher psychological distress (Gross and John, 2003a,b; Moore et al., 2008). Our study provides additional insight by showing that expressive suppression may hinder the development or utilization of cognitive flexibility, thereby indirectly exacerbating negative emotional experiences. This finding extends existing literature by emphasizing cognitive flexibility as a critical pathway through which expressive suppression can negatively influence teachers’ emotional health and underscores the importance of addressing such maladaptive strategies within teacher training and psychological interventions.

Additionally, the significant negative association between cognitive flexibility and anxiety and depression further highlights cognitive flexibility as an essential psychological resource for preschool teachers. This result is consistent with recent empirical studies identifying cognitive flexibility as a core psychological strength linked to resilience and emotional well-being in occupational contexts (Gao et al., 2025; Karbalaie et al., 2021; Motevalli et al., 2023). Our findings underscore cognitive flexibility’s protective function, suggesting that educators with greater cognitive flexibility may adapt more effectively to emotional challenges inherent in preschool environments. Thus, this study expands current theoretical frameworks by specifically validating cognitive flexibility as a mediator, enhancing our understanding of its protective mechanisms against negative emotional outcomes.

Finally, the differential effects of cognitive reappraisal and expressive suppression observed in this study emphasize the importance of distinguishing between emotion regulation strategies when designing interventions for teachers’ psychological health. Our results indicate that interventions aimed at promoting cognitive reappraisal and enhancing cognitive flexibility might significantly reduce negative emotions and bolster emotional resilience among preschool teachers. In contrast, efforts to discourage reliance on expressive suppression, given its potential negative impact on cognitive flexibility, might prove beneficial in protecting teachers’ mental health. Therefore, the current findings contribute practically by highlighting targeted areas for intervention, advancing both theoretical insights and evidence-based recommendations for improving preschool educators’ emotional well-being. Incorporate relevant courses or scenario-based simulations into pre-service teacher training programs to compare the effects of cognitive reappraisal versus expressive suppression. Pair these interventions with individual assessments using standardized scales, and establish dedicated funding to support kindergartens in introducing psychological supervision services.

Overall, our findings align closely with prior literature demonstrating cognitive reappraisal’s beneficial role and expressive suppression’s maladaptive impact on emotional health (Chervonsky and Hunt, 2019; Cutuli, 2014; Dryman and Heimberg, 2018; Gross and John, 2003a,b). However, subtle differences emerged concerning the direct effects of expressive suppression. Unlike certain previous studies reporting direct, robust associations between expressive suppression and elevated anxiety and depression (Dryman and Heimberg, 2018; Ehring et al., 2010; Gross and John, 2003a,b), our results showed minimal and statistically non-significant direct effects. We propose several possible explanations for this discrepancy. Firstly, our study explicitly incorporated cognitive flexibility as a mediator, utilizing structural equation modeling (SEM) to assess indirect relationships rather than solely focusing on direct associations. Previous studies primarily employed direct-effect models without explicitly evaluating mediation pathways. Therefore, our approach may have captured nuanced indirect influences previously overlooked, reducing or obscuring the direct relationships between expressive suppression and negative emotional outcomes. Secondly, the methodological rigor and statistical power achieved through a relatively large sample size (n = 392) and the application of advanced analytical methods (SEM with bootstrapping) may have provided greater accuracy and reliability in detecting indirect mediation pathways. Previous studies with smaller samples or simpler analytic approaches might have amplified or inflated the direct effects of expressive suppression.

Despite its contributions, several limitations should be acknowledged in the current study. Firstly, the cross-sectional nature of our research restricts causal interpretations regarding the relationships among emotion regulation strategies, cognitive flexibility, and negative emotional outcomes. Although our structural equation modeling provided robust evidence of mediation, longitudinal designs would further clarify the causal directions and temporal dynamics between these variables. Future studies employing longitudinal or experimental approaches could offer deeper insights into the stability and causal sequence of the observed mediation effects. Secondly, our data relied exclusively on self-report questionnaires, potentially introducing response biases such as social desirability and common-method variance. Future research could incorporate multi-method approaches, including observational assessments or physiological indicators of emotional regulation and stress responses, thereby enhancing methodological rigor and reducing potential biases inherent in self-report measures. Additionally, this study was conducted with preschool teachers in Beijing, which may limit the generalizability of findings to broader teacher populations or cultural contexts. Women accounted for 95.92% of the sample, which aligns with the gender distribution in the preschool education sector. The age range was concentrated between 21 and 35 years old (comprising 60.21% of the sample), and teaching experience spanned from less than 1 year to over 5 years, consistent with the youthful characteristics of kindergarten teachers in Beijing. However, the geographical scope was limited to Beijing, which may restrict the applicability of the findings to teachers in rural areas or other cities. Thus, future studies should examine similar mediation models across diverse cultural contexts and occupational groups to determine the extent to which these relationships generalize or differ cross-culturally. Finally, although cognitive flexibility emerged as a significant mediator, other psychological variables (e.g., emotional intelligence, resilience, social support) might concurrently mediate or moderate these relationships. Future research could explore these potential variables within a comprehensive model to deepen our understanding of the multiple pathways and boundary conditions influencing preschool teachers’ emotional health.

## Data Availability

The raw data supporting the conclusions of this article will be made available by the authors, without undue reservation.
